# Supplementary figures and images for: Activation of polycystin-1 signaling by binding of stalk-derived peptide agonists
Source: eLife. 2024 Oct 7;13:RP95992. doi: 10.7554/eLife.95992 (PMC11458180; doi:10.7554/eLife.95992)

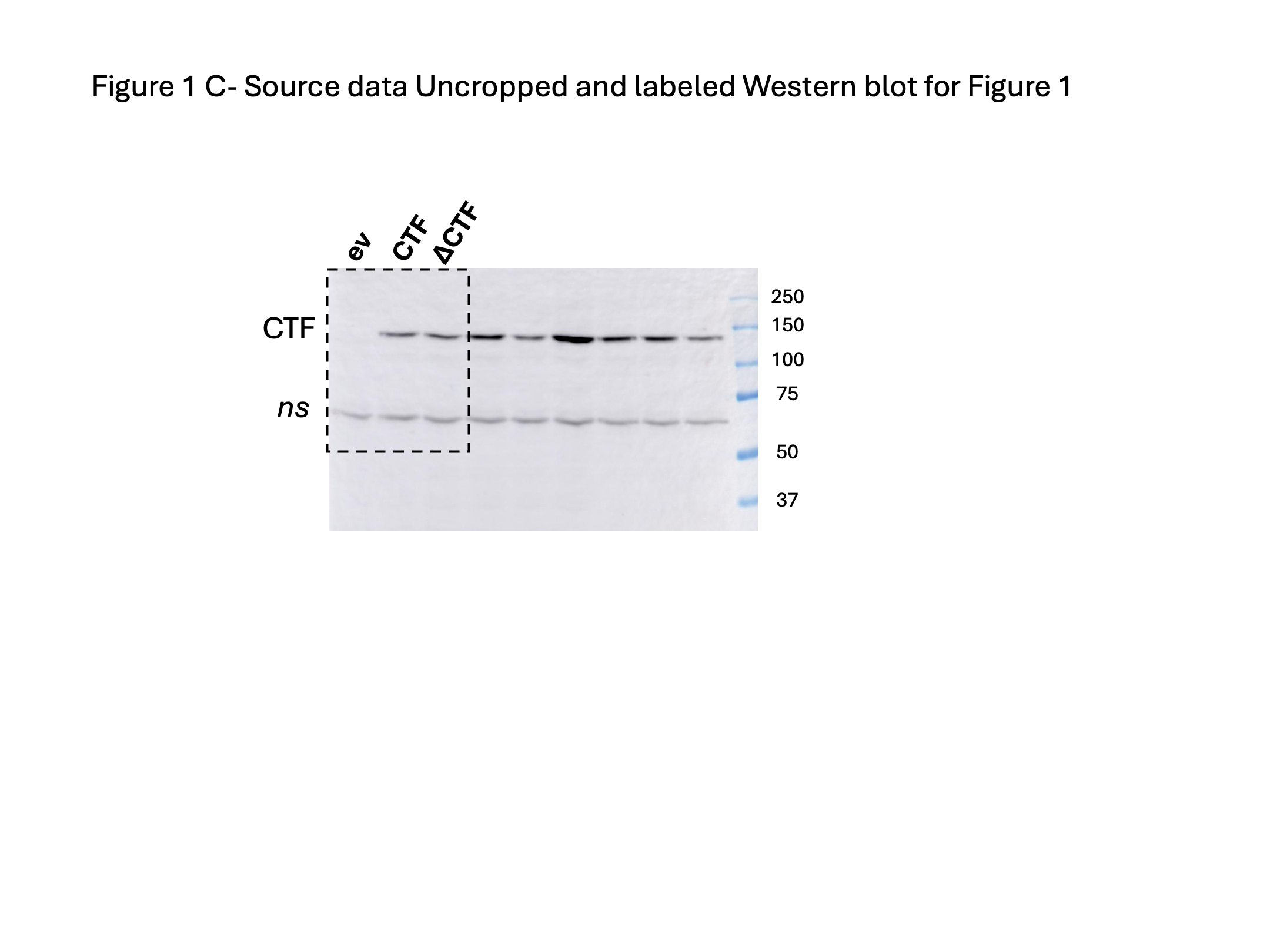

Supplement: Figure 1—source data 1. [file elife-95992-fig1-data1.zip › Figure 1- Source data 1 Uncropped and labeled Western blot for Fig 1C.tiff]

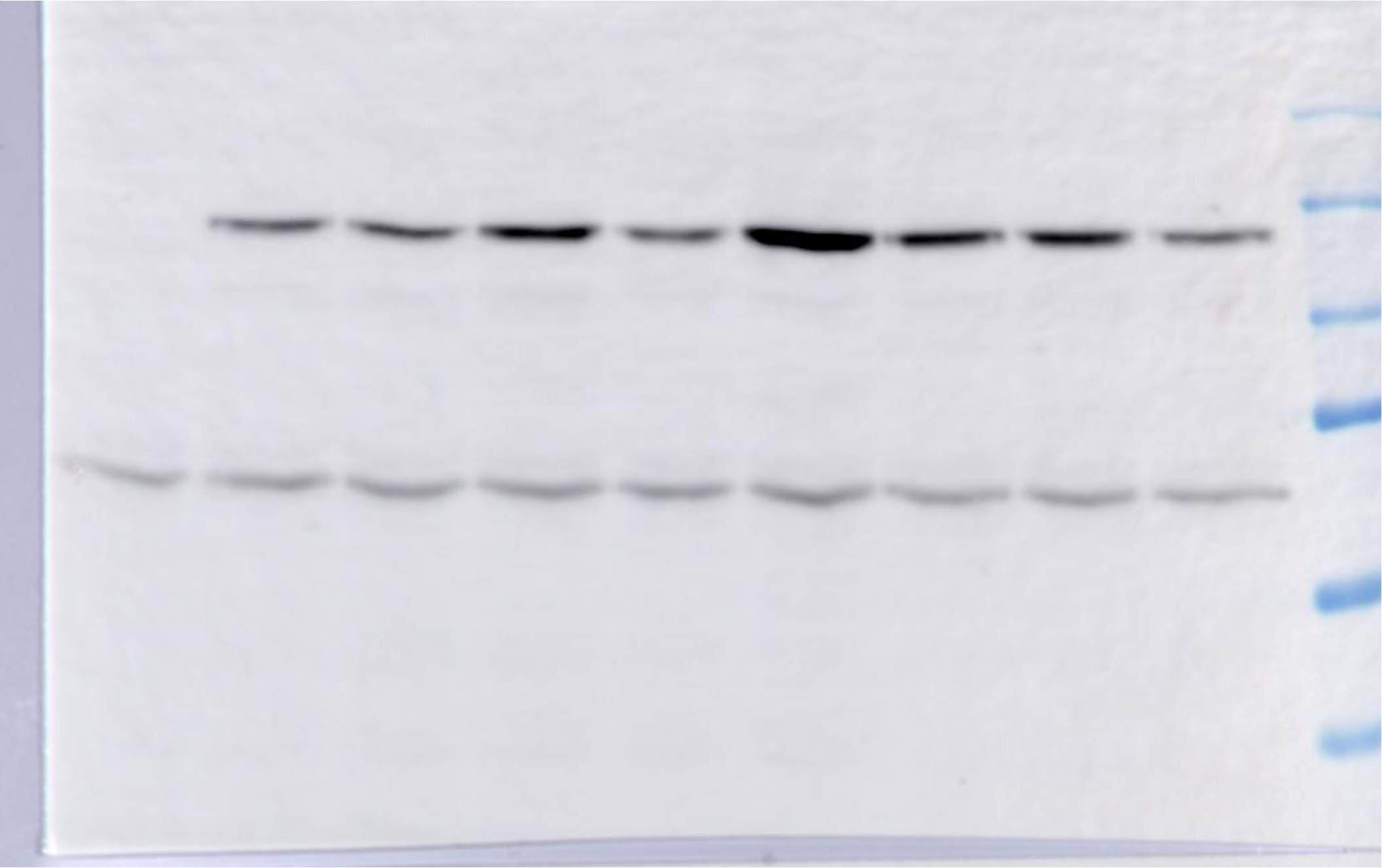

Supplement: Figure 1—source data 2. [file elife-95992-fig1-data2.zip › Figure 1-source data 1 Raw unedited blot for Figure 1C.tiff]

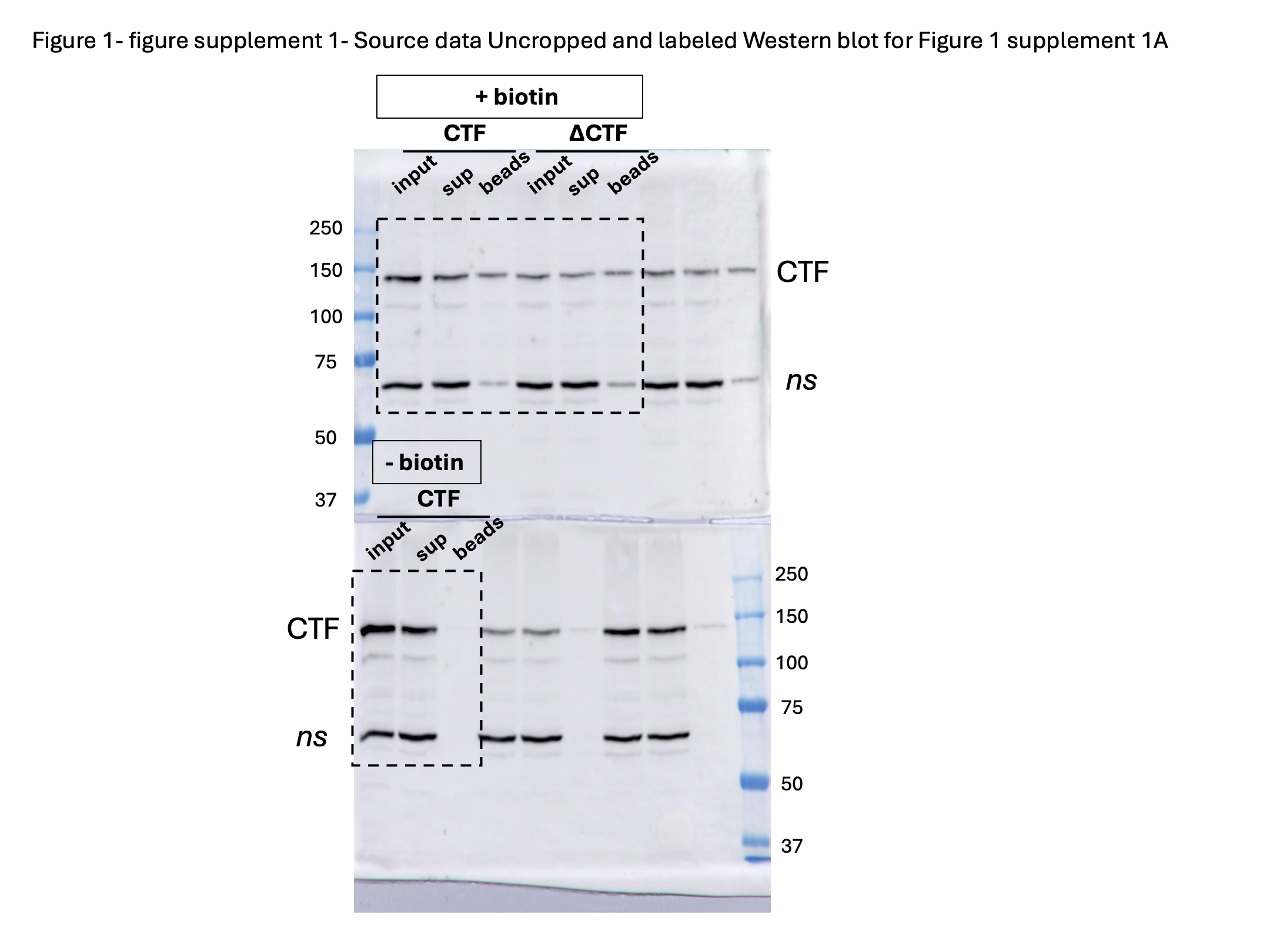

Supplement: Figure 1—figure supplement 1—source data 1. [file elife-95992-fig1-figsupp1-data1.zip › Figure 1-figure supplement 1-Source data 1 Uncropped and labeled Western.tiff]

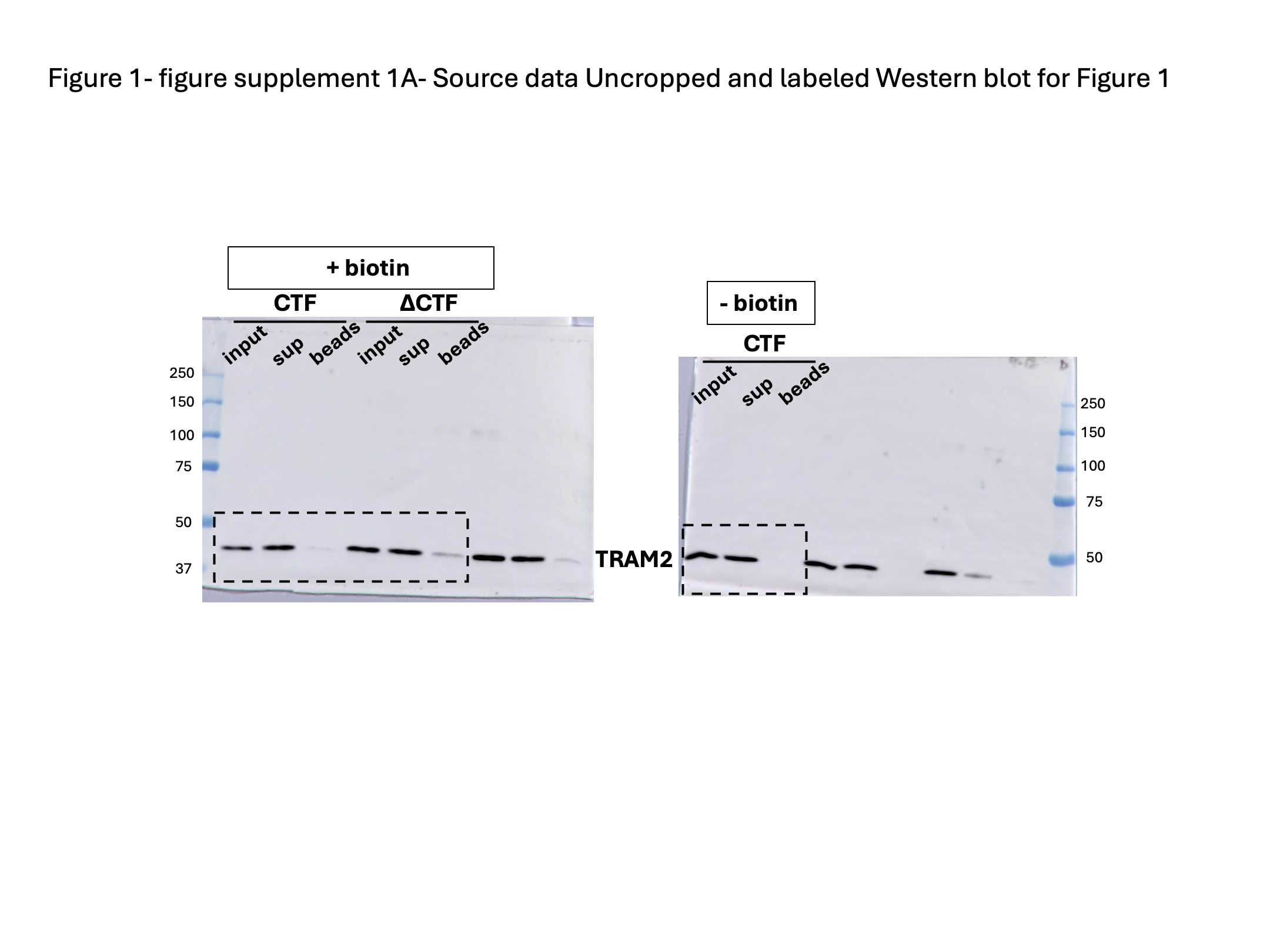

Supplement: Figure 1—figure supplement 1—source data 1. [file elife-95992-fig1-figsupp1-data1.zip › Figure 1-figure supplement 1-Source data 2 Uncropped and labeled Western blot.tiff]

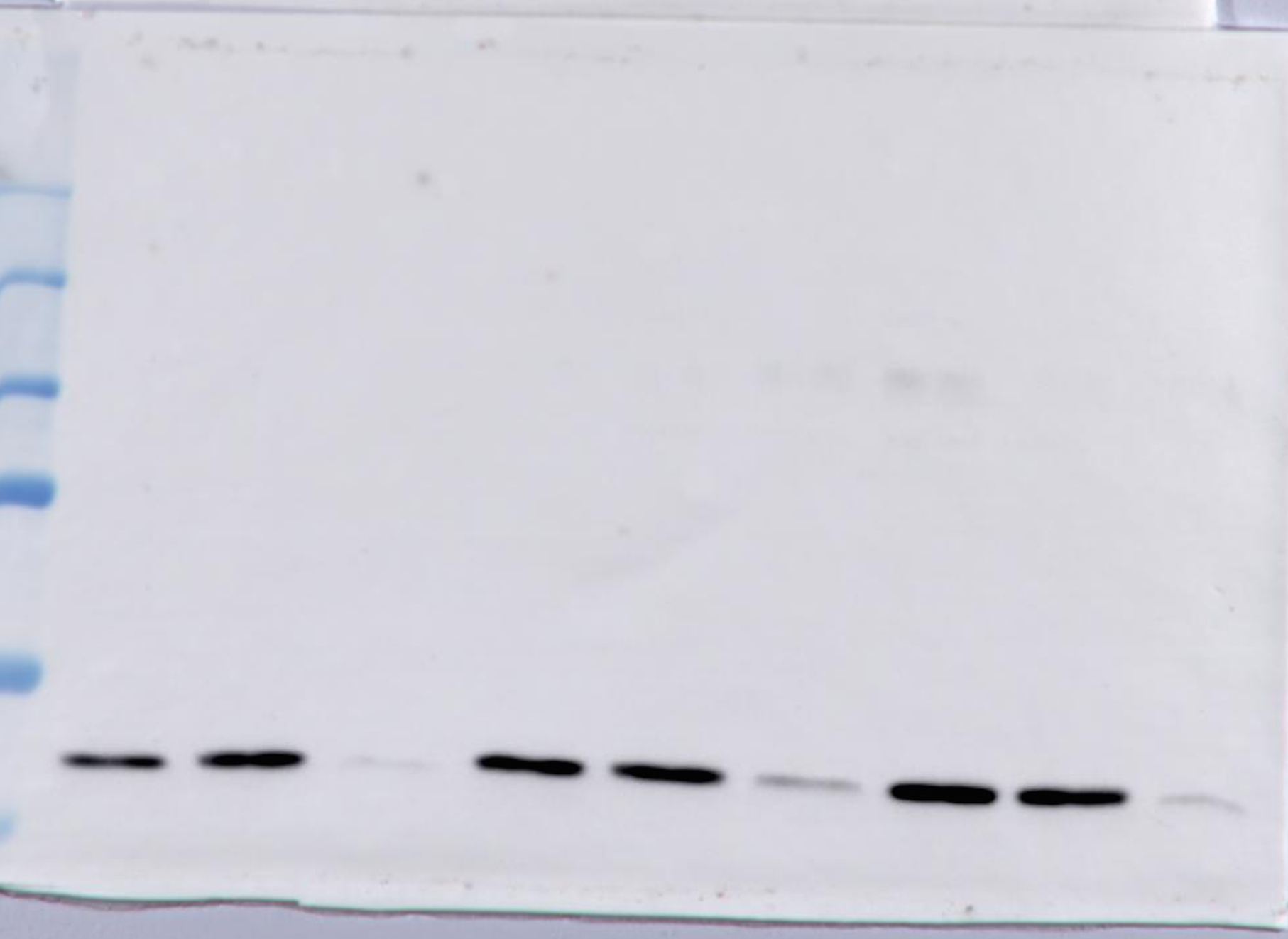

Supplement: Figure 1—figure supplement 1—source data 2. [file elife-95992-fig1-figsupp1-data2.zip › Figure 1 figure supplement 1-source data 2 Raw unedited blot for Figure 1 fig supp 1.tiff]

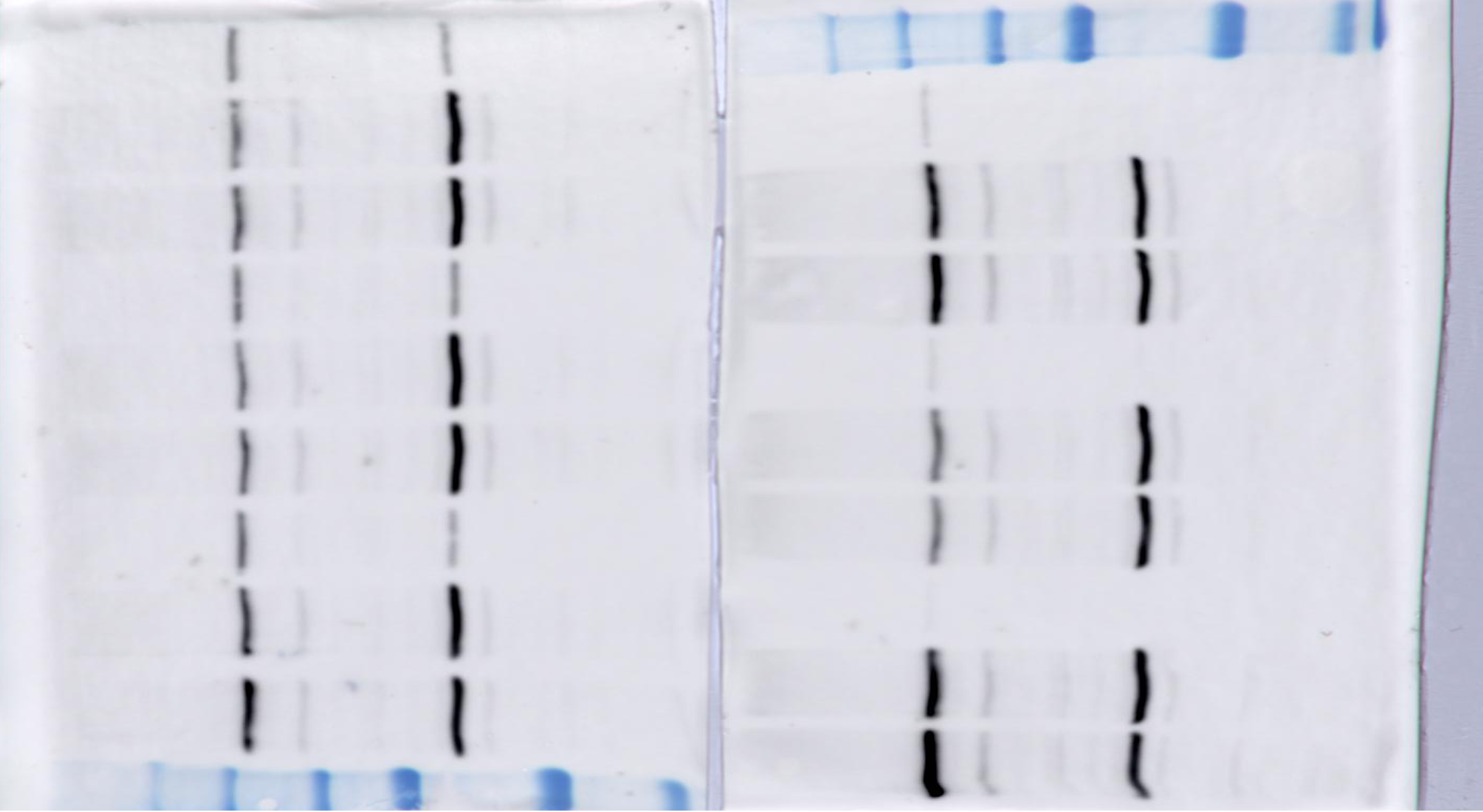

Supplement: Figure 1—figure supplement 1—source data 2. [file elife-95992-fig1-figsupp1-data2.zip › Figure 1 figure supplement 1-source data 1 Raw unedited blot for Figure 1 fig supp 1.tiff]

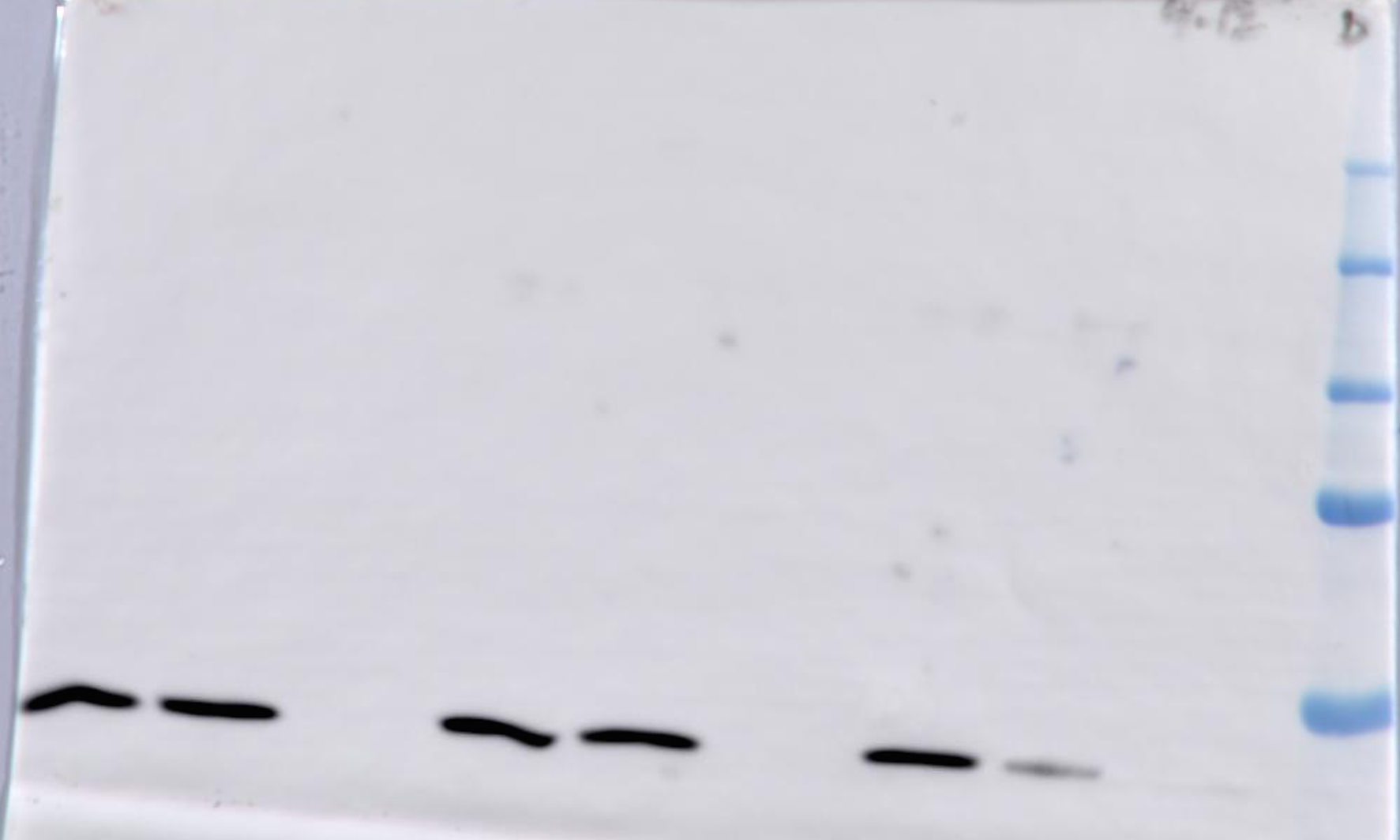

Supplement: Figure 1—figure supplement 1—source data 2. [file elife-95992-fig1-figsupp1-data2.zip › Figure 1 figure supplement 1-source data 3 Raw unedited blot for Figure 1 fig supp 1.tiff]
